# Low 25-hydroxyvitamin D levels and the risk of frailty syndrome: a systematic review and dose-response meta-analysis

**DOI:** 10.1186/s12877-018-0904-2

**Published:** 2018-09-04

**Authors:** Sang Yhun Ju, June Young Lee, Do Hoon Kim

**Affiliations:** 1 Department of Family Medicine, Yeouido St. Mary’s Hospital, College of Medicine, The Catholic University of Korea, 10, 63-Ro, Yeongdeungpo-Gu, Seoul, 07345 Republic of Korea; 2 Hospice Palliative Medicine, Division of Spirituality, Yeouido St. Mary’s Hospital, College of Medicine, The Catholic University of Korea, 10, 63-Ro, Yeongdeungpo-Gu, Seoul, 07345 Republic of Korea; 3 Department of Biostatistics, Korea University College of Medicine, 145, Anam-Ro, Seongbuk-Gu, Seoul, 02841 Republic of Korea; 4 Department of Family Medicine, Korea University Ansan Hospital, 70-9, Darigan 2-gil, Danwon-Gu, Ansan-Si, Gyeonggi-Do 15459 Republic of Korea

**Keywords:** Vitamin D, 25-hydroxyvitamin D, Frailty, Elderly, Cohort studies, Cross-sectional studies, Dose-response, Systematic review, Meta-analysis

## Abstract

**Background:**

Vitamin D deficiency and frailty are common with aging. Previous studies examining vitamin D status and frailty have produced mixed results, and in particular, the shape of the association has not been well established. We examined the association between 25-hydroxyvitamin D (25OHD) serum levels and frailty by performing a systematic review and dose-response meta-analysis.

**Methods:**

We searched the PubMed, EMBASE and Cochrane Library databases of Elsevier through February 2017. Cross-sectional and cohort studies that reported adjusted risk ratios with 95% confidence intervals (CI) for frailty with ≥3 categories of 25OHD serum levels were selected. Data extraction was performed independently by two authors. The reported risk estimates for 25OHD categories were recalculated, employing a comprehensive trend estimation from summarized dose-response data.

**Results:**

The pooled risk estimate of frailty syndrome per 25 nmol/L increment in serum 25OHD concentration was 0.88 (95% CI = 0.82–0.95, *I*^2^ = 86.8%) in the 6 cross-sectional studies and 0.89 (95% CI = 0.85–0.94, *I*^2^ = 0.0%) in the 4 prospective cohort studies. Based on the Akaike information criteria (AIC), a linear model was selected (AIC for the nonlinear model: − 5.4, AIC for the linear model: − 6.8 in the prospective cohort studies; AIC for the linear model: − 13.6, AIC for the nonlinear model: − 1.77 in the cross-sectional studies).

**Conclusions:**

This dose-response meta-analysis indicates that serum 25OHD levels are significantly and directly associated with the risk of frailty. Further studies should address the underlying mechanisms to explain this relationship and to determine whether vitamin D supplementation is effective for preventing frailty syndrome.

**Electronic supplementary material:**

The online version of this article (10.1186/s12877-018-0904-2) contains supplementary material, which is available to authorized users.

## Background

With increasing age, blood vitamin D concentrations decrease due to decreased kidney function, diminished sun exposure, intrinsic skin response to ultraviolet radiation and poor diet [1]. Vitamin D deficiency contributes to the development of osteoporosis and sarcopenia in older individuals, which increases the risk of fractures and falls and concomitant morbidity and mortality [2–4].

Frailty is a clinical state in which an individual’s vulnerability to developing increased dependency and/or mortality when exposed to a stressor is increased [5–7]. Numerous frailty diagnostic tools have been proposed, with one recent systematic review [8] identifying 67 various frailty instruments. The Physical Frailty Phenotype [7], which includes indicators such as shrinking, weakness, poor endurance, slowness, and low physical activity, is a widely used instrument for assessing physical frailty in the research setting. However, the concept of frailty, i.e., general vulnerability to various external stressors, extends far beyond the physical dimension, resulting in a multidimensional conceptualization of frailty based on interactions among various domains, including physical, psychological and social domains [5, 8–10]. Early detection of frailty may present an opportunity to introduce effective management strategies to improve outcomes [9].

An increasing number of studies investigating the association between 25-hydroxyvitamin D (25OHD) and frailty have yielded conflicting information. Although hypovitaminosis D can potentially increase the risk of frailty, not all observational studies have confirmed this relationship [11, 12]. Evidence from several cross-sectional studies supports a U-shaped [13] or linear inverse association between 25OHD levels and frailty [14, 15]. However, findings from longitudinal studies on the association between 25OHD levels and the development of frailty are inconsistent. Several studies have indicated that low vitamin D levels are significantly associated with frailty syndrome in the elderly, whereas others have found no association [16–19]. When the results were combined in a meta-analysis [12] published in 2016, the lowest 25OHD levels were associated with a 27% increase in the risk of frailty compared to the highest levels of 25OHD. However, the findings of the previous meta-analysis may be over- or underestimated due to variation in the 25OHD cutoff values used to define low and high 25OHD level categories as well as variation in the units used to measure serum levels of 25OHD.

Furthermore, the exact relationships, including whether a dose-response pattern exists, are currently unclear. Defining which levels of 25OHD are strongly associated with frailty syndrome is important for shaping elderly health recommendations about vitamin D supplementation considering the optimal serum 25OHD concentration. Furthermore, a recent prospective cohort study [20] in community-dwelling older women with a mean follow-up of 8.5 years did not identify a significant association between deficient (10–19 ng/mL) or insufficient (20–29.9 ng/mL) vitamin levels and incident frailty when compared to sufficient levels (≥30 ng/mL). Therefore, we conducted a systematic review and a dose-response meta-analysis of published cross-sectional and prospective cohort studies to further clarify the association between vitamin D and the risk of frailty.

## Methods

### Literature search

We searched the PubMed, Cochrane Library, and EMBASE databases via Elsevier through February 2017. A medical librarian together with the reviewers developed database-specific search strategies according to the particular subject headings and searching structure of the databases (Additional file 1). Furthermore, manual searches of the bibliographies of relevant articles were conducted to identify additional studies.

### Eligibility criteria

Studies were included in the meta-analysis if they met the following inclusion criteria: 1) an observational design including cross-sectional studies and cohort studies in humans, 2) the inclusion of frailty as a specified outcome, 3) a baseline assessment of serum 25OHD levels, 4) the inclusion of data on relative risk (RR) and its corresponding 95% confidence interval (CI) or data to calculate these values for frailty syndrome for each category of serum 25OHD level, and 5) the inclusion of the most recent and complete study (i.e., the most detailed category classification) if cohorts were duplicated in more than one study.

### Exclusion criteria, data extraction and quality assessment

Review articles, editorials, commentaries, and letters with no new data analysis, meta-analyses, and abstracts were excluded. The exclusion criteria for this study were as follows: 1) an experimental design was used, 2) the outcome was not frailty, and 3) only two serum 25OHD levels were specified. Two investigators (Sang Yhun Ju and Do Hoon Kim), coauthors of the present study, independently extracted the data from the original reports. The following information was extracted: the first author’s family name, year of publication, country of origin, the mean or median age of the participants, gender, sample size, the number of participants for each serum 25OHD level, the number of cases for each serum 25OHD concentration category, adjusted covariates, definitions of frailty used, the method of 25OHD assessment, follow-up duration, and categories of serum 25OHD and their corresponding RRs with their 95% CIs for frailty. The adjusted risk estimates that reflected the most comprehensive control were extracted to avoid potential confounding variables. Disagreements between the two reviewers were resolved by consensus. We planned, conducted, and reported this systematic review according to the widely accepted quality standards (Additional file 2) for reporting meta-analyses of observational studies in epidemiology [21].

### Statistical analysis

The methodology of the statistical analysis has been described in detail elsewhere [22]. In brief, the RR with 95% CI for each 25-nmol/L increase in the serum 25OHD in each study was calculated and was used for the meta-analysis. We performed a 2-stage random-effects dose-response meta-analysis to examine a potential nonlinear relationship between serum 25OHD levels and frailty [23]. We determined the best-fitting model, defined as the one with the smallest Akaike information criteria (AIC) [24]. The statistical heterogeneity of the studies was assessed using *I*^2^ statistics [25]. We regarded *I*^2^ values greater than 50% as indicators of high heterogeneity. The possibility of publication bias was assessed using Egger’s tests [26] and visual inspection of the funnel plot. We also applied the trim-and-fill algorithm [27] to identify and correct for funnel plot asymmetry. In the presence of publication bias, the *p* values for Egger’s tests were less than 0.1. All statistical analyses were performed using Stata software, version 14.0 (Stata Corp., College Station, TX, USA).

## Results

### Literature search and study selection

**Fig. 1:**
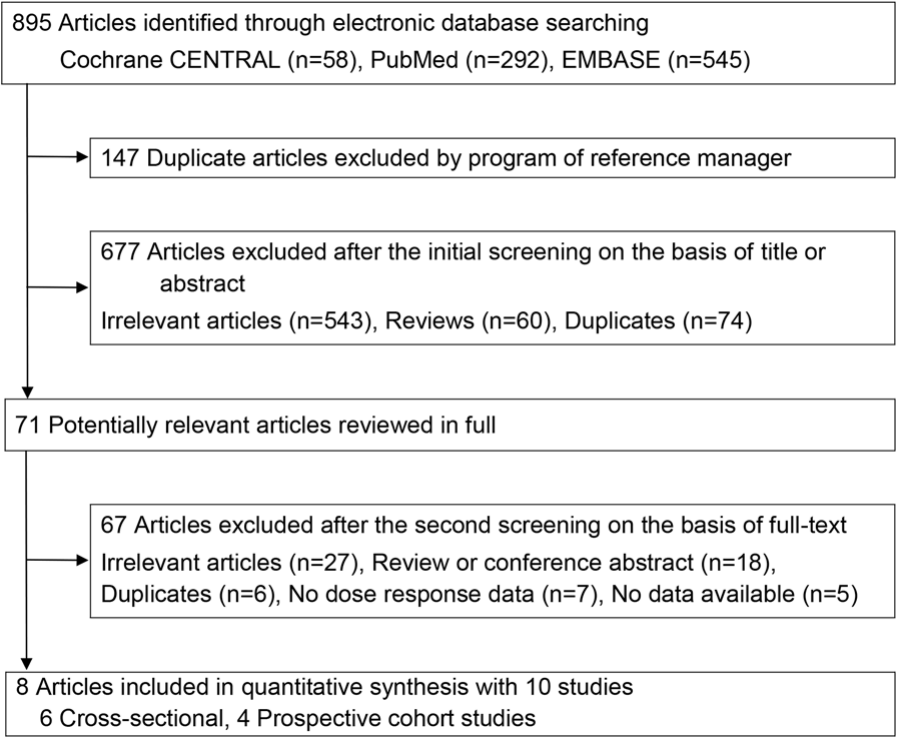
Flow diagram for the search strategy and study selection process

The process of identifying and selecting the studies is summarized in Fig. 1. A total of 895 articles were identified via Cochrane Central, PubMed, and EMBASE. Of these, 147 duplicate articles were excluded, and a further 677 articles were excluded based on their title and abstract, leaving 71 articles for further evaluation. After obtaining the full articles, we excluded a further 67 articles. Finally, we identified 8 articles including 10 studies that investigated the association between vitamin D status and frailty risk; 2 articles [16, 17] reported separate results for stratification by study design (i.e., cross-sectional and prospective cohort studies).

### Study characteristics and quality

Table 1 presents the information extracted from all included studies. Four studies had a cross-sectional design [13–15, 28], two were prospective studies [18, 20], and two studies reported both cross-sectional and prospective evaluations [16, 17]. All four studies were prospective cohort studies in a total of 8209 participants who were free of frailty at baseline. Among the participants, 737 incident cases of frailty occurred during a follow-up duration from 2.9 to 8.5 years. A total of six cross-sectional studies provided data on 20,949 participants, including 1802 cases of frailty. Five studies were conducted in Europe [15–18, 28], and the other three studies were conducted in the United States [13, 14, 28]. The mean age of the participants ranged from 62.2 to 79.2 years. Two studies [13, 28] included males only, two studies [14, 20] included females only, and four studies [16–18, 28] included both males and females. Most of the studies [13–15, 18, 20, 28] defined cases of frailty using frailty phenotypes, and the two studies by Puts et al. [16] and Schöttker et al. [17] used nine frailty indicators and the frailty index, respectively. Two studies [13, 14] used liquid chromatography tandem-mass spectrometry (LC-MS/MS), two studies [16, 18] used competitive binding protein assays, three studies [15, 17, 28] used immunoassays and one study [20] used radioreceptor assays. The selected studies reported their data on 25OHD levels in either nmol/L (three studies) [15–17] or ng/mL (five studies) [13, 14, 18, 20, 28]. We extracted the highest adjusted risk estimates from each study. Four studies adjusted for key covariates, including age, sex, timing of blood collection, BMI, smoking, and physical activity [13, 16, 18, 28]. The results of quality assessment are shown in Additional file 3. The average quality scores were 6.8 for the six cross-sectional studies and 8 for the four prospective cohort studies.

**Table 1:** Characteristics of studies and participants included in the meta-analysis of the association between serum 25-hydroxyvitamin D concentration and frailty

Author	Country	Gender	Participant (no.)	Case (no.)	Levels of 25OHD (Unit)	RR/OR (95% CI)	Definition (frailty) Assessment (25OHD)	Covariates
Year	Follow-up	Age range						Key-set covariates (yes/no)
Study name	(years)	(Mean age)						
Prospective cohort studies of serum 25-hydroxyvitamin D levels in relation to frailty								
Puts et al [16]	Netherlands	M and F	66	20	<25	1.90(0.92–3.95)	Nine Frailty indicators, competitive binding protein assay	^a^Key-sets of covariates, education, IL-6, CRP, alcohol, PTH, self-reported chronic disease, use of anti-inflammatory drugs, use of estrogen
2005	3,	>65	305	51	25-50	1.24 (0.77–2.00)		
LASA		(74.5, not frail; 79.2,frail)	514	54	>50	1.00 (Reference)		
					(nmol/L)			
Schöttker et al [17]	Germany	M and F	866	92	<30	1.18 (0.85–1.63)	Frailty Index, immunoassay	Age, sex, education, BMI, smoking, light physical activity and self-rated health
2014	8	50-74	2790	230	30-50	1.12 (0.88–1.43)		
ESTHER		(62.6)	2515	188	>50	1.00 (Reference)		
					(nmol/L)			
Vogt et al [18]	Germany	M and F	107	21	<15	2.84 (0.38–21.22)	Frailty Phenotype, competitive binding protein assay	^a^Key-sets of covariates, baseline frailty status, education, alcohol, CVD, diabetes, multimorbidity and PTH (yes)
2015	2.9	>65	100	4	15-20	0.46 (0.04–5.84)		
KORA		(75.5)	160	7	20-30	1.01 (0.1–10.07)		
			137	3	>30	1.00 (Reference)		
					(ng/mL)			
Buta et al [20]	United States	F	28	9	<10	2.29 (0.92–5.69)	Frailty Phenotype, radioreceptor assay	Age, race, education, smoking, season of blood draw, BMI, cardiovascular disease, diabetes mellitus, hyperlipidemia and hypertension
2017		70-79	135	29	10-19.9	1.44 (0.71–2.94)		
WHAS II	8.5	(73.8	141	21	20-29.9	1.08 (0.52–2.22)		
			65	8	>30	1.00 (Reference)		
					(ng/mL)			
Cross-sectional studies of serum 25-hydroxyvitamin D levels in relation to frailty								
Puts et al [16]	Netherlands	M and F	141	56	<25	2.55 (1.56–4.17)	Nine Frailty Indicators, competitive binding protein assay	^a^Key-sets of covariates, education, IL-6, CRP, alcohol, PTH, self-reported chronic disease, use of anti-inflammatory drugs, use of estrogen
2005		>65	471	166	25-50	1.66 (1.15–2.40)		
LASA		(74.5,not frail; 79.2,frail)	659	70	>50	1.00 (Reference)		
					(nmol/L)			
Ensrud et al [14]	United States	F	1280	301	<15	1.47 (1.19–1.82)	Frailty Phenotype, LC-MS/MS	^a^Key-sets of covariates, site, self-reported health status, education, alcohol, comorbidity, and short MMSE
2010		>65	1233	217	15-19.9	1.24 (0.99–1.54)		
SOF		(76.7)	2428	329	20.0-29.9	1.00 (Reference)		
			1366	218	>30	1.32 (1.06–1.63)		
					(ng/mL)			
Ensrud et al [13]	United States	M	408	54	<20	1.47 (1.07–2.02)	Frailty Phenotype, LC-MS/MS	Age, race, site, season of blood draw, BMI, self-reported health status, education, living alone, smoking status, alcohol intake, comorbidity score, Teng 3MS score, and baseline frailty status
2011		>65	803	55	20-29.9	1.02 (0.78–1.32)		
MrOS		(73.8)	395	21	>30	1.00 (Reference)		
					(ng/mL)			
Tajar et al [15]	Italy, belgium, Poland, Sweden, UK, Spain, Hungary and Estonia	M	524	43	<50	5.74 (2.12–15.6)	Frailty Phenotype, radioimmunoassay	Age, centre, smoking, co-morbid conditions and PTH
2013		≥60	453	19	50-75	3.55 (1.27–9.90)		
EMAS		(69.5)	399	7	>75	1.00 (Reference)		
					(nmol/L)			
Schöttker et al [17]	Germany	M and F	1444	52	<30	1.90 (1.30–2.78)	Frailty Index, immunoassay	Age, sex, education, BMI, smoking, light physical activity and self-rated health
2014		50-74	4199	120	30-50	1.48 (1.09–2.01)		
ESTHER		(62.2)	3936	87	>50	1.00 (Reference)		
					(nmol/L)			
Pabst et al [28]	Germany	M and F	292	17	<15	1.00 (Reference)	Frailty Phenotype, enhanced chemiluminescence immunoassay	^a^Key-sets of covariates, years of education, self-perceived economic situation, co-morbidity score, MMSE
2015		65-90	192	8	15-20	0.71 (0.22–2.32)		
KORA-Age		(75.6)	257	12	20-30	0.60 (0.19–1.91)		
					(ng/mL)			

Abbreviations: *BMI* body mass index, *CRP* C-reactive protein, *ESTHER* Epidemiological investigations of the chances of preventing, recognizing early and optimally treating chronic diseases in an elderly population, *KORA* Cooperative Health Research in the Region of Augsburg, *IL-6* interleukin-6, *LASA* Longitudinal Aging Study Amsterdam, *LC-MS/MS* liquid chromatography tandem-mass spectrometry, *MrOS* Osteoporotic Fractures in Men Study, *PTH* parathyroid hormone, *SOF* Study of Osteoporotic Fractures, *WHAS II* Women’s Health and Aging Study II, *25(OH)D* 25-hydroxyvitamin D. ^a^Key-sets of covariates: age, sex, season of blood draw, body mass index (or obesity), smoking, and physical activity

**Fig. 2:**
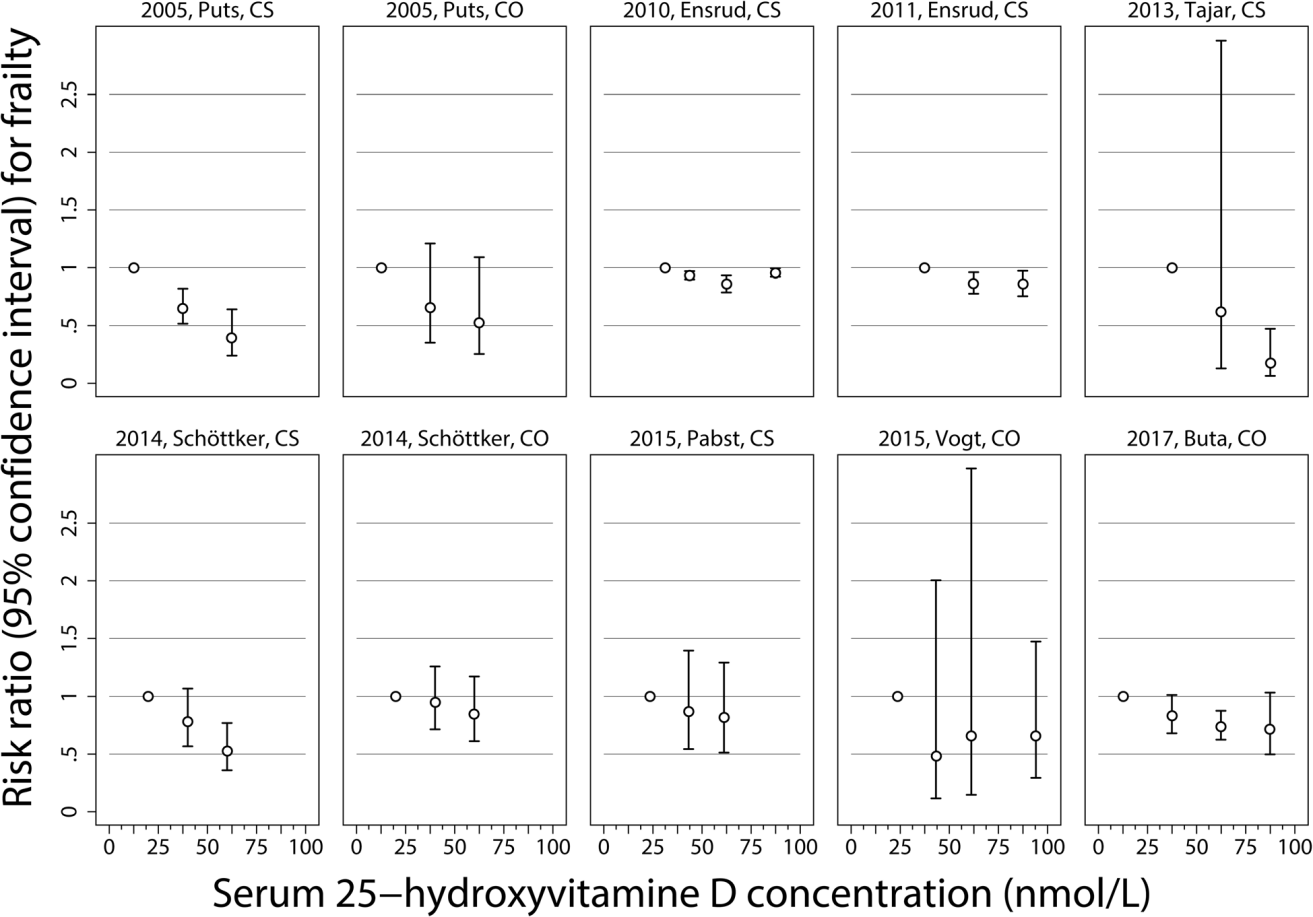
Study-specific risk ratios and 95% confidence intervals of frailty syndrome risk according to study-specific serum levels of 25-hydroxyvitamin D (25OHD). Depending on available information, the median, midpoints or means of the categories were used for defining study-specific serum levels of 25OHD (nmol/L). CS, cross-sectional study; CO, prospective cohort studies

The reported risk estimates for the association between 25OHD level intervals and frailty are illustrated in Fig. 2. A roughly inverse linear relationship was found between 25OHD levels and frailty risk in most studies, with the exception of the cross-sectional study by Ensrud et al. [14], which identified a U-shaped association, and the cohort study by Vogt et al. [18], which found no association between 25OHD levels and frailty risk. In all the other studies, the group with the highest 25OHD levels had the lowest frailty risk.

### Quantitative data synthesis

**Fig. 3:**
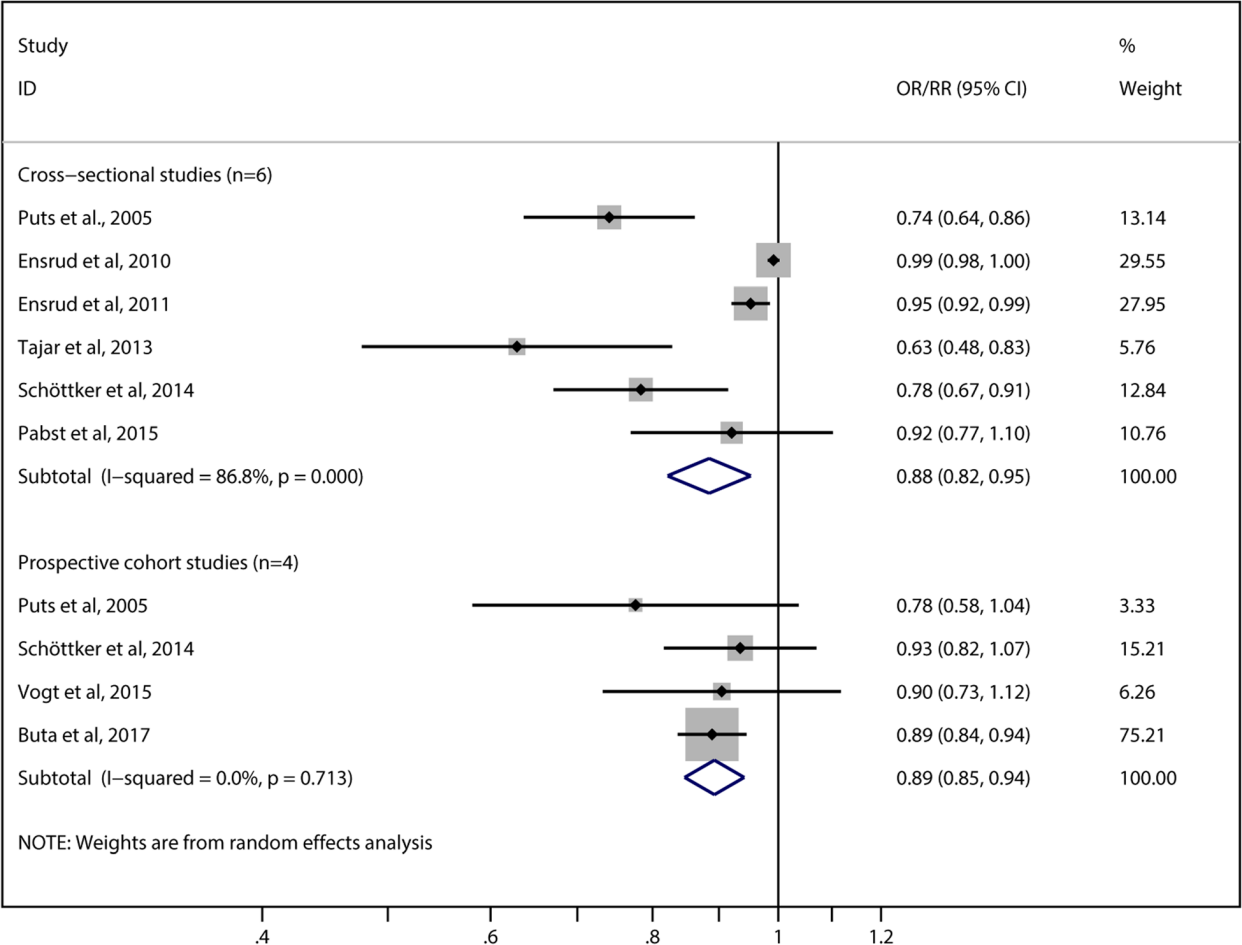
Forest plots of the risk ratios (RRs) of frailty syndrome per 25 nmol/L increment in serum 25-hydroxyvitamin D concentration using a random-effects analysis. The squares represent study-specific RR (the square sizes are proportional to the weight of each study in the overall estimate); the horizontal lines represent 95% confidence intervals (CIs), and the diamond represents the overall RR estimate with 95% CI

The reported effect estimates of the 25OHD groups were converted in the risk estimates for a 25-nmol/L increase in 25OHD levels and pooled for the meta-analysis. The meta-analysis summarized the results of six cross-sectional studies with 21,207 participants including 1802 cases of frailty and four prospective cohort studies accounting for a total of 8746 individuals and 864 frailty events during follow-up. 25OHD was significantly inversely associated with frailty in four of six cross-sectional studies and one of four cohort studies. The pooled risk estimates of frailty syndrome per 25-nmol/L increment in serum 25OHD concentration were 0.88 (95% CI = 0.82–0.95, *I*^2^ = 86.8%) in the six cross-sectional studies and 0.89 (95% CI = 0.85–0.94, *I*^2^ = 0.0%) in the four prospective cohort studies (Fig. 3).

For comparisons with results from other studies and reviews, the RRs for the 50 and 75 nmol/L increases in 25OHD levels were also estimated: 0.65 (95% CI = 0.50–0.85) and 0.52 (95% CI = 0.35–0.78), respectively, for cross-sectional studies and 0.76 (95% CI = 0.66–0.87) and 0.66 (0.53–0.82), respectively, for prospective cohort studies. The funnel plot of the linear dose-response slopes was somewhat asymmetric for the cross-sectional studies, with smaller studies tending to have larger risk estimates, suggesting publication bias. A publication bias was detected with Egger’s test for the cross-sectional studies (Additional file 4: Figure S1; *p* = 0.007), but not for the prospective cohort studies (Additional file 4: Figure S2; *P* = 0.693). The trim-and-fill sensitivity method imputed estimates from three hypothesized negative unpublished estimates. The corrected effect estimate for a 25-nmol/L increase in the 25OHD level was reduced to 0.98 (95% CI = 0.90–1.05, *I*^2^ = 88.9%), demonstrating no relationship between the risk of frailty and serum 25OHD levels after accounting for a potential publication bias.

**Fig. 4:**
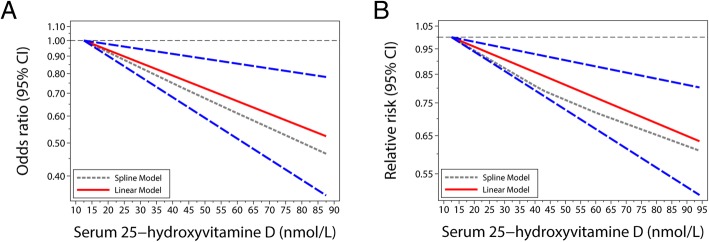
Risk ratios (RRs) and the corresponding 95% confidence intervals (CIs) for the dose-response relationship between serum 25-hydroxyvitamin D concentrations (nmol/L) and risk of frailty syndrome among the populations. **a**, cross-sectional studies; **b**, prospective cohort studies. The solid and long-dashed lines represent the estimated RRs and their 95% CIs, respectively. The short-dashed line represents the non-linear relationship

Four prospective cohort studies were included in the restricted cubic spline models (Fig. 4a). Based on the AIC, a linear model was selected (AIC for the nonlinear model: − 5.4, AIC for the linear model: − 6.8). Compared with a serum 25OHD level of 12.5 nmol/L, the RR (95% CI) for frailty was 0.96 (0.94–0.98) for 20 nmol/L, 0.86 (0.80–0.94) for 40 nmol/L, 0.77 (0.67–0.88) for 60 nmol/L, and 0.64 (0.50–0.80) for 94 nmol/L. Six cross-sectional studies were included in the restricted cubic spline models (Fig. 4b). Based on the AIC, a linear model was selected (AIC for the linear model: − 13.6, AIC for the nonlinear model: − 1.77). Compared with the serum 25OHD level of 12.5 nmol/L, the OR (95% CI) of frailty for the cross-sectional studies was 0.94 (0.90–0.98) for 20 nmol/L, 0.85 (0.77–0.94) for 31 nmol/L, 0.78 (0.68–0.91) for 40 nmol/L, 0.66 (0.52–0.86) for 60 nmol/L, and 0.52 (0.35–0.78) for 94 nmol/L.

### Subgroup analyses

Subgroup analyses were performed based on geographic location (Europe vs. United States, definition frailty, key sets of covariates (yes vs. no), Newcastle-Ottawa Scale (≥7 vs < 7), and follow-up years (≥median vs. <median). The subgroups are presented in Table 2. Overall, an inverse association between a 25 nmol/L increase in 25OHD levels and risk of frailty was consistently observed in each subgroup. In the subgroup analyses of cross-sectional studies, the inverse association was statistically significant for the geographic region of Europe (OR = 0.78, 95% CI = 0.68–0.88, *I*^2^ = 50.6%), the subgroup (nine frailty indicators and the frailty index) of frailty definition (OR = 0.76, 95% CI = 0.68–0.85, *I*^2^ = 0%), the subgroup of other 25OHD measurement methods (OR = 0.78, 95% CI = 0.68–0.88, *I*^2^ = 50.6%) and the Newcastle-Ottawa scale ≥7 subgroup (OR = 0.71–0.91, *I*^2^ = 41.4). In addition, within the prospective cohort studies, we found that the association was significant for phenotype definition of frailty (RR = 0.89, 95% CI = 0.84–0.94, *I*^2^ = 0%), duration of follow-up ≥5.6 year (RR = 0.90, 95% CI = 0.85–0.95, *I*^2^ = 0%) and the subgroup (yes) of key-set covariates (RR = 0.90, 95% CI = 0.85–0.95, *I*^2^ = 0%).

**Table 2:** Frailty risk per 25 nmol/L increase in serum 25-hydroxyvitamin D in subgroup meta-analyses of the cross-sectional studies and the prospective cohort studies

Group	Subgroup	No.	OR/RR (95% CI)	*I*^*2*^ (%)	*P*
Cross-sectional studies					
Geographic location	Europe	4	0.78 (0.68–0.88)	50.6	0.108
	United States	2	0.98 (0.94–1.01)	79.8	0.026
Definition of frailty	Phenotype	4	0.95 (0.89–1.01)	81.1	0.001
	Others	2	0.76 (0.68–0.85)	0.0	0.612
Method of ^a^25OHD assessment	^b^LC-MS/MS	2	0.98 (0.94–1.01)	79.8	0.026
	Others	4	0.78 (0.68–0.88)	50.6	0.108
^c^Key-sets of covariates	Yes	3	0.87 (0.75–1.02)	80.1	0.007
	No	3	0.81 (0.63–1.04)	89.6	< 0.001
^d^Newcastle-Ottawa Scale	≥ 7	3	0.81 (0.71–0.91)	41.4	0.182
	< 7	3	0.95 (0.95–1.02)	86.9	< 0.001
Prospective cohort studies					
Definition of frailty	Phenotype	2	0.89 (0.84–0.94)	0.0	0.877
	Others	2	0.89 (0.76–1.04)	22.8	0.255
Follow-up (years)	≥ 5.6	2	0.90 (0.85–0.95)	0.0	0.510
	< 5.6	2	0.86 (0.72–1.02)	0.0	0.403
^c^Key-sets of covariates	Yes	2	0.90 (0.85–0.95)	0.0	0.510
	No	2	0.86 (0.72–1.02)	0.0	0.403

^a^*25OHD* 25-hydroxyvitmain D, ^b^*LC-MS/MS* liquid chromatography tandem-mass spectrometry ^c^Key-sets of covariates: age, sex, season of blood draw, body mass index (or obesity), smoking, and physical activity; ^d^Newcastle-Ottawa Scale: Total score could range from 0 to 9

## Discussion

To our knowledge, this is the first dose-response quantitative systematic review of observational studies investing the effect of serum 25OHD levels on the risk of frailty using data from both cross-sectional and prospective cohort studies. This meta-analysis of data from more than 20,000 study participants demonstrates a statistically significant inverse association between serum 25OHD levels and the risk of frailty, and this finding was consistent across subgroups. A 25-nmol/L increase in 25OHD levels was associated with an 11% decrease in the incidence of frailty in prospective cohort studies and a 12% decrease in the risk of frailty in the cross-sectional studies. A statistically linear relationship between serum 25OHD levels and the risk of frailty was also found, even after adjustments for other known risk factors.

We estimated a protective effect of a 25-nmol/L increase in 25OHD levels against frailty because this number is the difference between the upper limit of vitamin D deficiency, defined by most experts as a 25OHD level less than 50 nmol/L, and the lower limit of sufficient vitamin D levels above 75 nmol/L. Additionally, a daily intake of at least 1000 IU (25 μg/d; 1 μg = 40 IU) of vitamin D3 appears to be required to elevate vitamin D concentrations by 25 nmol/L, which would ensure that no less than 50% of the population has the recommended 25OHD level of at least 75 nmol/L [3, 18]. Several studies indicated increases in serum 25OHD levels of only ~ 7–10 nmol/L per 400 IU of daily vitamin D supplementation [29, 30]. Recently, a study reported that the extent of the 25OHD increase upon vitamin D supplementation depended on 25OHD baseline levels, age, and body weight [31]. Hence, a new guideline for the dosing requirement for vitamin supplementation in frail elderly persons, based on initial vitamin D levels, should be established after further investigation.

A previous meta-analysis [12] of seven prospective cohorts reported an inverse association between vitamin D deficiency and frailty. However, only data in the highest compared with the lowest categories of 25OHD were used rather than the use of all categories. This dose response meta-analysis allowed us to evaluate risk across the entire spectrum of observed 25OHD levels. We observed an inverse linear association between 25OHD levels and the risk of frailty among the elderly population, suggesting that any incremental increase in serum 25OHD level was associated with a decreased risk of frailty. However, because serum 25OHD concentrations in the current data for the frailty study range from 12.5 to 95 nmol/L, we have not been able to investigate the dose-response relationship between higher levels of 25OHD, i.e., > 95 nmol/L and risk of frailty. It is too early to determine whether there is a specific cutoff level of serum 25OHD that increases or reduces the risk of developing frailty syndrome because a limited number of studies used serum 25OHD levels as a categorical variable and provided RR data for each category and because of the variation in the definition of frailty. Thus, the results of this analysis should be interpreted cautiously. We cannot rule out the possibility that the serum 25OHD level has a threshold rather than dose-response effect on the risk of frailty.

Our results suggest that high 25OHD levels are associated with a lower risk of frailty in elderly people. Conversely, frailty itself may contribute to lower 25OHD by reducing the levels of outdoor activity and sunlight exposure. Elderly individuals with frailty are at a high risk of developing vitamin D deficiency due to decreased dietary intake, less sun exposure, and a decreased capacity to produce sufficient amounts of calcitriol due to an age-related decline in hydroxylation by the kidney [1]. The causality of the association between low vitamin D levels and the frailty syndrome has not been completely elucidated. Nevertheless, there are several potential biological mechanisms that could explain the inverse association between vitamin D and frailty.

Considerable overlap exists between sarcopenia and frailty, especially in terms of the physical aspects of the frailty phenotype: low grip strength, gait speed and muscle mass [32]. While the underlying mechanisms and pathophysiology of sarcopenia remain to be clarified, inadequate nutritional intake in older individuals may contribute to the multifactorial pathogenesis of sarcopenia [33]. In particular, vitamin D, one of the most popular micronutrients, was reported to play important roles in muscle differentiation, stimulation of calcium and phosphorus transport and muscle contraction [34]. A muscle biopsy study revealed atrophy of type II muscle fibers in subjects with profound vitamin D deficiency [35]. During sudden movement, the fast and strong type II muscle fibers are the first to be recruited to avoid falling [36]. A meta-analysis observed that daily vitamin D doses in the range of 700 to 1000 IU or achieving serum concentrations between 60 and 95 nmol/L reduced the risk of falling by 19% in older individuals [3]. 1,25-Dihydroxyvitamin D (1,25OHD) can act on muscle fibers by binding to its nuclear vitamin D receptor (VDR) and thereby increasing the de novo synthesis of protein, which regulates muscle strength [36]. VDR number decreases with aging in several organs involved in calcium metabolism, and 1alpha-hydroxylase activity decreases mainly due to a decrease in renal function, reducing vitamin D activation [1]. An age-related decline in VDR expression is supported by studies in rats in which VDR expression declined with advancing age in both the intestine and bone [37, 38]. When 25OHD levels are low, active metabolite 1,25OHD levels and calcium absorption decrease [39]. This reduced serum calcium led to an increase in parathyroid hormone levels to stimulate 1,25OHD production, resulting in an increased risk of bone turnover and bone loss [39, 40]. Consequently, a decline in muscle function and strength caused by vitamin D could explain slowness, low physical activity and weakness.

The last pathway through which low vitamin D may affect frailty is related to its hypothesized anti-inflammatory properties [41]. Several studies [16, 17, 29] have demonstrated a heightened inflammatory state among frail older adults marked by high serum levels of inflammatory mediators, such as cytokines and acute phase proteins, supporting the existence of a dysregulated immune system in frailty. An increased susceptibility to infection and risk of autoimmune disease has been shown in 25OHD deficiency. Illness may be the beginning of a vicious cycle between 25OHD deficiency and frailty [5]. Individuals with illness tend to exhibit poor nutritional status, go outside less frequently and experience less sun exposure, which are underlying causes of low serum 25OHD concentrations [5, 6, 10, 33]. A recent study suggested that 1,25OHD may be an important regulator of the inflammatory response during bacterial infection [42]. Active vitamin D metabolites can downregulate inflammatory markers via the nuclear VDR expressed in antigen-presenting cells, and vitamin D deficiency may result in increased pro-inflammatory cytokines that impact muscle strength and performance [43, 44].

The findings of our study should be interpreted within its limitations. The included studies have no data regarding vitamin D supplementations evaluated through food frequency questionnaires or self-administered questionnaires, which could affect serum 25OHD concentrations; therefore, the present study may under- or overestimate our results. The definitions for frailty used in the included studies were different (i.e., Fried phenotype, modified phenotype, nine frailty indications, and frailty index), thus affecting our pooled analysis, although subgroup analysis was performed according to definitions of frailty. Because several eligible studies did not provide sufficient information for a dose-response analysis of 25OHD levels, the number of participants, cases, and logarithms of RRs and corresponding standard errors, we excluded the potential related studies [19, 45–49], which may introduce a potential selection bias in our analysis. Unlike those observed for prospective cohort studies, the results from cross-sectional studies were somewhat heterogeneous but consistently pointed to an inverse relationship despite the observation that the strength of the association differed substantially across studies. Additionally, publication bias seems to have occurred for cross-sectional but not for prospective cohort studies in the literature, which may contribute to the stronger inverse association observed among the former. Our meta-analysis only included studies published in English and did not search for unpublished studies that might contribute to the asymmetrical funnel plot. When we explored the influence of a potential publication bias trim-and-fill method, our findings revealed no significant association of 25OHD with frailty among the cross-sectional studies. However, detection and adjustment of publication bias is difficult and somewhat controversial when only a small number of trials is available [50]. The funnel plot suggests the presence of three negative, outlying studies that were not balanced by positive studies. Additional investigations including a reference review did not reveal any further peer-reviewed studies for inclusion. Although this may represent publication bias, it may also reflect a truly significant inverse relationship between vitamin D status and frailty.

## Conclusion

Our findings suggest that 25OHD serum levels are independently associated with the risk of frailty, which is consistent with the results of a nonlinear analysis and a linear regression analysis. Further interventional research should investigate whether vitamin D supplementation can be useful for preventing frailty in the elderly population.

## Additional files

Additional file 1: Search strategy. (DOCX 24 kb)

Additional file 2: MOOSE Checklist for Meta-analyses of Observational Studies. (DOCX 55 kb)

Additional file 3: Quality of the observational studies in the meta-analysis based on the Newcastle-Ottawa Scale. (DOCX 14 kb)

Additional file 4: Publication bias for association between serum 25-hydroxyvitamin D concentration per 25-nmol/L increment and frailty syndrome. **Figure S1**. Begg’s Funnel plot with 95% confidence intervals in the meta-analysis of the cross-sectional studies. **Figure S2**. Begg’s Funnel plot with 95% confidence intervals in the meta-analysis of the prospective cohort studies. (DOCX 59 kb)

## Abbreviations

1,25OHD: 1,25-dihydroxyvitamin D
25OHD: 25-hydroxyvitamin D
AIC: Akaike information criteria
CI: Confidence interval
OR: Odds ratio
RR: Relative risk
