# Supplementary material for: Low 25-hydroxyvitamin D levels and the risk of frailty syndrome: a systematic review and dose-response meta-analysis
Source: BMC Geriatr. 2018 Sep 4;18:206. doi: 10.1186/s12877-018-0904-2 (PMC6124011; doi:10.1186/s12877-018-0904-2)
Supplement: Supplementary file 1 — Search strategy. (DOCX 24 kb) [file 12877_2018_904_MOESM1_ESM.docx]

**Additional File 1: Search strategy**

| **Database** | **Search** | **Search terms** | **Hits** |
| --- | --- | --- | --- |
| **PubMed** | #1 | "Vitamin D"[Mesh] | 49,540 |
|  | #2 | "Vitamin D"[TW] | 57,868 |
|  | #3 | "Cholecalciferol"[Mesh] | 24,035 |
|  | #4 | "Cholecalciferol"[TW] OR “Calciol"[TW] OR “(3 beta,5Z,7E)-9,10-Secocholesta-5,7,10(19)-trien-3-ol"[TW] OR “Vitamin D 3"[TW] OR “Vitamin D3"[TW] OR “Cholecalciferols"[TW] | 13,784 |
|  | #5 | "25-Hydroxyvitamin D 2"[Mesh] | 739 |
|  | #6 | "25-Hydroxyvitamin D 2"[TW] OR “25 Hydroxyvitamin D 2"[TW] OR “25-Hydroxyergocalciferol"[TW] OR “25 Hydroxyergocalciferol"[TW] OR “25-Hydroxyvitamin D2"[TW] OR “25 Hydroxyvitamin D2"[TW] OR “9,10-Secoergosta-5,7,10(19),22-tetraene-3 beta,25-diol"[TW] OR “Ercalcidiol"[TW] OR “25-Hydroxycalciferol"[TW] OR “25 Hydroxycalciferol"[TW] | 887 |
|  | #7 | "Vitamin D Deficiency"[Mesh] | 23,200 |
|  | #8 | "Vitamin D Deficiency"[TW] OR “Deficiency, Vitamin D"[TW] OR “Deficiencies, Vitamin D"[TW] OR “Vitamin D Deficiencies"[TW] | 15,449 |
|  | #9 | "25-hydroxyvitamin D" [Supplementary Concept] | 4,903 |
|  | #10 | "25-hydroxyvitamin D"[TW] | 10,682 |
|  | #11 | "Avitaminosis"[Mesh] | 66,407 |
|  | #12 | "Avitaminosis"[TW] OR “Avitaminoses"[TW] OR “Deficiency, Vitamin"[TW] OR “Deficiencies, Vitamin"[TW] OR “Vitamin Deficiencies"[TW] OR “Vitamin Deficiency"[TW] | 4,177 |
|  | #13 | "Calcitriol"[Mesh] | 13,249 |
|  | #14 | "Calcitriol"[TW] OR “1 alpha,25-Dihydroxyvitamin D3"[TW] OR “1 alpha,25 Dihydroxyvitamin D3"[TW] OR “D3, 1 alpha,25-Dihydroxyvitamin"[TW] OR “alpha,25-Dihydroxyvitamin D3, 1"[TW] OR “1,25-Dihydroxyvitamin D3"[TW] OR “1,25 Dihydroxyvitamin D3"[TW] OR “D3, 1,25-Dihydroxyvitamin"[TW] OR “1 alpha,25-Dihydroxycholecalciferol"[TW] OR “1 alpha,25 Dihydroxycholecalciferol"[TW] OR “1,25-Dihydroxycholecalciferol"[TW] OR “1,25 Dihydroxycholecalciferol"[TW] OR “Bocatriol"[TW] OR “Leo Brand of Calcitriol"[TW] OR “Calcitriol Leo Brand"[TW] OR “Calcijex"[TW] OR “Abbott Brand of Calcitriol"[TW] OR “Calcitriol Abbott Brand"[TW] OR “Calcitriol KyraMed"[TW] OR “KyraMed, Calcitriol"[TW] OR “KyraMed Brand of Calcitriol"[TW] OR “Calcitriol KyraMed Brand"[TW] OR “Calcitriol-Nefro"[TW] OR “Calcitriol Nefro"[TW] OR “CalcitriolNefro"[TW] OR “Medice Brand of Calcitriol"[TW] OR “Calcitriol Medice Brand"[TW] OR “Decostriol"[TW] OR “Jenapharm Brand of Calcitriol"[TW] OR “Calcitriol Jenapharm Brand"[TW] OR “MC1288"[TW] OR “MC-1288"[TW] OR “MC 1288"[TW] OR “Osteotriol"[TW] OR “Gry Brand of Calcitriol"[TW] OR “Calcitriol Gry Brand"[TW] OR “Renatriol"[TW] OR “RenaCare Brand of Calcitriol"[TW] OR “Calcitriol RenaCare Brand"[TW] OR “Rocaltrol"[TW] OR “Roche Brand of Calcitriol"[TW] OR “Calcitriol Roche Brand"[TW] OR “Hoffmann-La Roche Brand of Calcitriol"[TW] OR “Hoffmann La Roche Brand of Calcitriol"[TW] OR “Silkis"[TW] OR “Galderma Brand of Calcitriol"[TW] OR “Calcitriol Galderma Brand"[TW] OR “Sitriol"[TW] OR “Alphapharm Brand of Calcitriol"[TW] OR “Calcitriol Alphapharm Brand"[TW] OR “Soltriol"[TW] OR “Tirocal"[TW] OR “Cryopharma Brand of Calcitriol"[TW] OR “Calcitriol Cryopharma Brand"[TW] OR “20-epi-1alpha,25-dihydroxycholecaliferol"[TW] OR “20 epi 1alpha,25 dihydroxycholecaliferol"[TW] OR “1,25-dihydroxy-20-epi-Vitamin D3"[TW] OR “1,25 dihydroxy 20 epi Vitamin D3"[TW] OR “D3, 1,25-dihydroxy-20-epi-Vitamin"[TW] OR “1,25(OH)2-20epi-D3"[TW] OR “1 alpha, 25-dihydroxy-20-epi-Vitamin D3"[TW] OR “1 alpha, 25 dihydroxy 20 epi Vitamin D3"[TW] | 20,961 |
|  | **#15 Combine** | **((((((((((((("Vitamin D"[Mesh]) OR "Vitamin D"[TW]) OR "Cholecalciferol"[Mesh]) OR (("Cholecalciferol"[TW] OR “Calciol"[TW] OR “(3 beta,5Z,7E)-9,10-Secocholesta-5,7,10(19)-trien-3-ol"[TW] OR “Vitamin D 3"[TW] OR “Vitamin D3"[TW] OR “Cholecalciferols"[TW]))) OR "25-Hydroxyvitamin D 2"[Mesh]) OR (("25-Hydroxyvitamin D 2"[TW] OR “25 Hydroxyvitamin D 2"[TW] OR “25-Hydroxyergocalciferol"[TW] OR “25 Hydroxyergocalciferol"[TW] OR “25-Hydroxyvitamin D2"[TW] OR “25 Hydroxyvitamin D2"[TW] OR “9,10-Secoergosta-5,7,10(19),22-tetraene-3 beta,25-diol"[TW] OR “Ercalcidiol"[TW] OR “25-Hydroxycalciferol"[TW] OR “25 Hydroxycalciferol"[TW]))) OR "Vitamin D Deficiency"[Mesh]) OR (("Vitamin D Deficiency"[TW] OR “Deficiency, Vitamin D"[TW] OR “Deficiencies, Vitamin D"[TW] OR “Vitamin D Deficiencies"[TW]))) OR "25-hydroxyvitamin D" [Supplementary Concept]) OR "25-hydroxyvitamin D"[TW]) OR "Avitaminosis"[Mesh]) OR (("Avitaminosis"[TW] OR “Avitaminoses"[TW] OR “Deficiency, Vitamin"[TW] OR “Deficiencies, Vitamin"[TW] OR “Vitamin Deficiencies"[TW] OR “Vitamin Deficiency"[TW]))) OR "Calcitriol"[Mesh]) OR (("Calcitriol"[TW] OR “1 alpha,25-Dihydroxyvitamin D3"[TW] OR “1 alpha,25 Dihydroxyvitamin D3"[TW] OR “D3, 1 alpha,25-Dihydroxyvitamin"[TW] OR “alpha,25-Dihydroxyvitamin D3, 1"[TW] OR “1,25-Dihydroxyvitamin D3"[TW] OR “1,25 Dihydroxyvitamin D3"[TW] OR “D3, 1,25-Dihydroxyvitamin"[TW] OR “1 alpha,25-Dihydroxycholecalciferol"[TW] OR “1 alpha,25 Dihydroxycholecalciferol"[TW] OR “1,25-Dihydroxycholecalciferol"[TW] OR “1,25 Dihydroxycholecalciferol"[TW] OR “Bocatriol"[TW] OR “Leo Brand of Calcitriol"[TW] OR “Calcitriol Leo Brand"[TW] OR “Calcijex"[TW] OR “Abbott Brand of Calcitriol"[TW] OR “Calcitriol Abbott Brand"[TW] OR “Calcitriol KyraMed"[TW] OR “KyraMed, Calcitriol"[TW] OR “KyraMed Brand of Calcitriol"[TW] OR “Calcitriol KyraMed Brand"[TW] OR “Calcitriol-Nefro"[TW] OR “Calcitriol Nefro"[TW] OR “CalcitriolNefro"[TW] OR “Medice Brand of Calcitriol"[TW] OR “Calcitriol Medice Brand"[TW] OR “Decostriol"[TW] OR “Jenapharm Brand of Calcitriol"[TW] OR “Calcitriol Jenapharm Brand"[TW] OR “MC1288"[TW] OR “MC-1288"[TW] OR “MC 1288"[TW] OR “Osteotriol"[TW] OR “Gry Brand of Calcitriol"[TW] OR “Calcitriol Gry Brand"[TW] OR “Renatriol"[TW] OR “RenaCare Brand of Calcitriol"[TW] OR “Calcitriol RenaCare Brand"[TW] OR “Rocaltrol"[TW] OR “Roche Brand of Calcitriol"[TW] OR “Calcitriol Roche Brand"[TW] OR “Hoffmann-La Roche Brand of Calcitriol"[TW] OR “Hoffmann La Roche Brand of Calcitriol"[TW] OR “Silkis"[TW] OR “Galderma Brand of Calcitriol"[TW] OR “Calcitriol Galderma Brand"[TW] OR “Sitriol"[TW] OR “Alphapharm Brand of Calcitriol"[TW] OR “Calcitriol Alphapharm Brand"[TW] OR “Soltriol"[TW] OR “Tirocal"[TW] OR “Cryopharma Brand of Calcitriol"[TW] OR “Calcitriol Cryopharma Brand"[TW] OR “20-epi-1alpha,25-dihydroxycholecaliferol"[TW] OR “20 epi 1alpha,25 dihydroxycholecaliferol"[TW] OR “1,25-dihydroxy-20-epi-Vitamin D3"[TW] OR “1,25 dihydroxy 20 epi Vitamin D3"[TW] OR “D3, 1,25-dihydroxy-20-epi-Vitamin"[TW] OR “1,25(OH)2-20epi-D3"[TW] OR “1 alpha, 25-dihydroxy-20-epi-Vitamin D3"[TW] OR “1 alpha, 25 dihydroxy 20 epi Vitamin D3"[TW]))** | **125,106** |
|  | #16 | "frailty"[TW] | 7,001 |
|  | #17 | "Frail Elderly"[Mesh] | 8,364 |
|  | #18 | "Frail Elderly"[TW] OR “Elderly, Frail"[TW] OR “Frail Elders"[TW] OR “Elder, Frail"[TW] OR “Elders, Frail"[TW] OR “Frail Elder"[TW] OR “Functionally-Impaired Elderly"[TW] OR “Elderly, Functionally-Impaired"[TW] OR “Functionally Impaired Elderly"[TW] OR “Frail Older Adults"[TW] OR “Adult, Frail Older"[TW] OR “Adults, Frail Older"[TW] OR “Frail Older Adult"[TW] OR “Older Adult, Frail"[TW] OR “Older Adults, Frail"[TW] | 9,952 |
|  | **#19 Combine** | **(("frailty"[TW]) OR "Frail Elderly"[Mesh]) OR (("Frail Elderly"[TW] OR “Elderly, Frail"[TW] OR “Frail Elders"[TW] OR “Elder, Frail"[TW] OR “Elders, Frail"[TW] OR “Frail Elder"[TW] OR “Functionally-Impaired Elderly"[TW] OR “Elderly, Functionally-Impaired"[TW] OR “Functionally Impaired Elderly"[TW] OR “Frail Older Adults"[TW] OR “Adult, Frail Older"[TW] OR “Adults, Frail Older"[TW] OR “Frail Older Adult"[TW] OR “Older Adult, Frail"[TW] OR “Older Adults, Frail"[TW]))** | **14,315** |
|  | **#20 Combine** | **#15 AND #19** | **292** |
|  |  |  |  |
|  |  |  |  |
| **Database** | **Search** | **Search terms** | **Hits** |
| **EMBASE** | #1 | vitamin d'/exp OR 'vitamin d' | 130,797 |
|  | #2 | colecalciferol'/exp OR 'colecalciferol' | 18,612 |
|  | #3 | cholecalciferol'/exp OR 'cholecalciferol' OR 'calciol'/exp OR 'calciol' OR '(3 beta,5z,7e)-9,10-secocholesta-5,7,10(19)-trien-3-ol' OR 'vitamin d 3'/exp OR 'vitamin d 3' OR 'vitamin d3'/exp OR 'vitamin d3' OR 'cholecalciferols'/exp OR 'cholecalciferols' | 25,080 |
|  | #4 | 25 hydroxyergocalciferol'/exp OR '25 hydroxyergocalciferol' | 953 |
|  | #5 | 25-hydroxyvitamin d 2'/exp OR '25-hydroxyvitamin d 2' OR '25 hydroxyvitamin d 2'/exp OR '25 hydroxyvitamin d 2' OR '25-hydroxyergocalciferol'/exp OR '25-hydroxyergocalciferol' OR '25-hydroxyvitamin d2'/exp OR '25-hydroxyvitamin d2' OR '25 hydroxyvitamin d2'/exp OR '25 hydroxyvitamin d2' OR '9,10-secoergosta-5,7,10(19),22-tetraene-3 beta,25-diol' OR 'ercalcidiol'/exp OR 'ercalcidiol' OR '25-hydroxycalciferol'/exp OR '25-hydroxycalciferol' OR '25 hydroxycalciferol'/exp OR '25 hydroxycalciferol' | 1,048 |
|  | #6 | vitamin d deficiency'/exp OR 'vitamin d deficiency' | 23,985 |
|  | #7 | deficiency, vitamin d' OR 'deficiencies, vitamin d' OR 'vitamin d deficiencies' | 327 |
|  | #8 | 25 hydroxyvitamin d'/exp OR '25 hydroxyvitamin d' | 19,323 |
|  | #9 | vitamin deficiency'/exp OR 'vitamin deficiency' | 65,043 |
|  | #10 | avitaminosis'/exp OR 'avitaminosis' OR 'avitaminoses' OR 'deficiency, vitamin'/exp OR 'deficiency, vitamin' OR 'deficiencies, vitamin' OR 'vitamin deficiencies'/exp OR 'vitamin deficiencies' | 65,183 |
|  | #11 | calcitriol'/exp OR 'calcitriol' | 29,296 |
|  | #12 | calcitriol'/exp OR 'calcitriol' OR '1 alpha,25-dihydroxyvitamin d3' OR '1 alpha,25 dihydroxyvitamin d3' OR 'd3, 1 alpha,25-dihydroxyvitamin' OR 'alpha,25-dihydroxyvitamin d3, 1' OR '1,25-dihydroxyvitamin d3'/exp OR '1,25-dihydroxyvitamin d3' OR '1,25 dihydroxyvitamin d3'/exp OR '1,25 dihydroxyvitamin d3' OR 'd3, 1,25-dihydroxyvitamin' OR '1 alpha,25-dihydroxycholecalciferol' OR '1 alpha,25 dihydroxycholecalciferol' OR '1,25-dihydroxycholecalciferol'/exp OR '1,25-dihydroxycholecalciferol' OR '1,25 dihydroxycholecalciferol'/exp OR '1,25 dihydroxycholecalciferol' OR 'bocatriol'/exp OR 'bocatriol' OR 'leo brand of calcitriol' OR 'calcitriol leo brand' OR 'calcijex'/exp OR 'calcijex' OR 'abbott brand of calcitriol' OR 'calcitriol abbott brand' OR 'calcitriol kyramed' OR 'kyramed, calcitriol' OR 'kyramed brand of calcitriol' OR 'calcitriol kyramed brand' OR 'calcitriol-nefro' OR 'calcitriol nefro' OR 'calcitriolnefro' OR 'medice brand of calcitriol' OR 'calcitriol medice brand' OR 'decostriol'/exp OR 'decostriol' OR 'jenapharm brand of calcitriol' OR 'calcitriol jenapharm brand' OR 'mc1288' OR 'mc-1288'/exp OR 'mc-1288' OR 'mc 1288'/exp OR 'mc 1288' OR 'osteotriol'/exp OR 'osteotriol' OR 'gry brand of calcitriol' OR 'calcitriol gry brand' OR 'renatriol'/exp OR 'renatriol' OR 'renacare brand of calcitriol' OR 'calcitriol renacare brand' OR 'rocaltrol'/exp OR 'rocaltrol' OR 'roche brand of calcitriol' OR 'calcitriol roche brand' OR 'hoffmann-la roche brand of calcitriol' OR 'hoffmann la roche brand of calcitriol' OR 'silkis'/exp OR 'silkis' OR 'galderma brand of calcitriol' OR 'calcitriol galderma brand' OR 'sitriol'/exp OR 'sitriol' OR 'alphapharm brand of calcitriol' OR 'calcitriol alphapharm brand' OR 'soltriol'/exp OR 'soltriol' OR 'tirocal'/exp OR 'tirocal' OR 'cryopharma brand of calcitriol' OR 'calcitriol cryopharma brand' OR '20-epi-1alpha,25-dihydroxycholecaliferol' OR '20 epi 1alpha,25 dihydroxycholecaliferol' OR '1,25-dihydroxy-20-epi-vitamin d3' OR '1,25 dihydroxy 20 epi vitamin d3' OR 'd3, 1,25-dihydroxy-20-epi-vitamin' OR '1,25(oh)2-20epi-d3' OR '1 alpha, 25-dihydroxy-20-epi-vitamin d3' OR '1 alpha, 25 dihydroxy 20 epi vitamin d3' | 29,970 |
|  | **#13 Combine** | **vitamin d'/exp OR 'vitamin d' OR 'colecalciferol'/exp OR 'colecalciferol' OR 'cholecalciferol'/exp OR 'cholecalciferol' OR 'calciol'/exp OR 'calciol' OR '(3 beta,5z,7e)-9,10-secocholesta-5,7,10(19)-trien-3-ol' OR 'vitamin d 3'/exp OR 'vitamin d 3' OR 'vitamin d3'/exp OR 'vitamin d3' OR 'cholecalciferols'/exp OR 'cholecalciferols' OR '25 hydroxyergocalciferol'/exp OR '25 hydroxyergocalciferol' OR '25-hydroxyvitamin d 2'/exp OR '25-hydroxyvitamin d 2' OR '25 hydroxyvitamin d 2'/exp OR '25 hydroxyvitamin d 2' OR '25-hydroxyergocalciferol'/exp OR '25-hydroxyergocalciferol' OR '25-hydroxyvitamin d2'/exp OR '25-hydroxyvitamin d2' OR '25 hydroxyvitamin d2'/exp OR '25 hydroxyvitamin d2' OR '9,10-secoergosta-5,7,10(19),22-tetraene-3 beta,25-diol' OR 'ercalcidiol'/exp OR 'ercalcidiol' OR '25-hydroxycalciferol'/exp OR '25-hydroxycalciferol' OR '25 hydroxycalciferol'/exp OR '25 hydroxycalciferol' OR 'vitamin d deficiency'/exp OR 'vitamin d deficiency' OR 'deficiency, vitamin d' OR 'deficiencies, vitamin d' OR 'vitamin d deficiencies' OR '25 hydroxyvitamin d'/exp OR '25 hydroxyvitamin d' OR 'vitamin deficiency'/exp OR 'vitamin deficiency' OR 'avitaminosis'/exp OR 'avitaminosis' OR 'avitaminoses' OR 'deficiency, vitamin'/exp OR 'deficiency, vitamin' OR 'deficiencies, vitamin' OR 'vitamin deficiencies'/exp OR 'vitamin deficiencies' OR 'calcitriol'/exp OR 'calcitriol' OR 'calcitriol'/exp OR 'calcitriol' OR '1 alpha,25-dihydroxyvitamin d3' OR '1 alpha,25 dihydroxyvitamin d3' OR 'd3, 1 alpha,25-dihydroxyvitamin' OR 'alpha,25-dihydroxyvitamin d3, 1' OR '1,25-dihydroxyvitamin d3'/exp OR '1,25-dihydroxyvitamin d3' OR '1,25 dihydroxyvitamin d3'/exp OR '1,25 dihydroxyvitamin d3' OR 'd3, 1,25-dihydroxyvitamin' OR '1 alpha,25-dihydroxycholecalciferol' OR '1 alpha,25 dihydroxycholecalciferol' OR '1,25-dihydroxycholecalciferol'/exp OR '1,25-dihydroxycholecalciferol' OR '1,25 dihydroxycholecalciferol'/exp OR '1,25 dihydroxycholecalciferol' OR 'bocatriol'/exp OR 'bocatriol' OR 'leo brand of calcitriol' OR 'calcitriol leo brand' OR 'calcijex'/exp OR 'calcijex' OR 'abbott brand of calcitriol' OR 'calcitriol abbott brand' OR 'calcitriol kyramed' OR 'kyramed, calcitriol' OR 'kyramed brand of calcitriol' OR 'calcitriol kyramed brand' OR 'calcitriol-nefro' OR 'calcitriol nefro' OR 'calcitriolnefro' OR 'medice brand of calcitriol' OR 'calcitriol medice brand' OR 'decostriol'/exp OR 'decostriol' OR 'jenapharm brand of calcitriol' OR 'calcitriol jenapharm brand' OR 'mc1288' OR 'mc-1288'/exp OR 'mc-1288' OR 'mc 1288'/exp OR 'mc 1288' OR 'osteotriol'/exp OR 'osteotriol' OR 'gry brand of calcitriol' OR 'calcitriol gry brand' OR 'renatriol'/exp OR 'renatriol' OR 'renacare brand of calcitriol' OR 'calcitriol renacare brand' OR 'rocaltrol'/exp OR 'rocaltrol' OR 'roche brand of calcitriol' OR 'calcitriol roche brand' OR 'hoffmann-la roche brand of calcitriol' OR 'hoffmann la roche brand of calcitriol' OR 'silkis'/exp OR 'silkis' OR 'galderma brand of calcitriol' OR 'calcitriol galderma brand' OR 'sitriol'/exp OR 'sitriol' OR 'alphapharm brand of calcitriol' OR 'calcitriol alphapharm brand' OR 'soltriol'/exp OR 'soltriol' OR 'tirocal'/exp OR 'tirocal' OR 'cryopharma brand of calcitriol' OR 'calcitriol cryopharma brand' OR '20-epi-1alpha,25-dihydroxycholecaliferol' OR '20 epi 1alpha,25 dihydroxycholecaliferol' OR '1,25-dihydroxy-20-epi-vitamin d3' OR '1,25 dihydroxy 20 epi vitamin d3' OR 'd3, 1,25-dihydroxy-20-epi-vitamin' OR '1,25(oh)2-20epi-d3' OR '1 alpha, 25-dihydroxy-20-epi-vitamin d3' OR '1 alpha, 25 dihydroxy 20 epi vitamin d3'** | **174,457** |
|  | #14 | frailty'/exp OR frailty | 10,323 |
|  | #15 | frail elderly'/exp OR 'frail elderly' | 9,826 |
|  | #16 | elderly, frail' OR 'frail elders' OR 'elder, frail' OR 'elders, frail' OR 'frail elder' OR 'functionally-impaired elderly' OR 'elderly, functionally-impaired' OR 'functionally impaired elderly' OR 'frail older adults' OR 'adult, frail older' OR 'adults, frail older' OR 'frail older adult' OR 'older adult, frail' OR 'older adults, frail' | 1,419 |
|  | **#17 Combine** | **frailty'/exp OR frailty OR 'frail elderly'/exp OR 'frail elderly' OR 'elderly, frail' OR 'frail elders' OR 'elder, frail' OR 'elders, frail' OR 'frail elder' OR 'functionally-impaired elderly' OR 'elderly, functionally-impaired' OR 'functionally impaired elderly' OR 'frail older adults' OR 'adult, frail older' OR 'adults, frail older' OR 'frail older adult' OR 'older adult, frail' OR 'older adults, frail'** | **17,961** |
|  | **#18 Combine** | **#13 AND #17** | **593** |
|  | **#19 Limit** | **#13 AND #17 AND [embase]/lim** | **545** |
|  |  |  |  |
|  |  |  |  |
| **Database** | **Search** | **Search terms** | **Hits** |
| **Cochrane** | #1 | [mh "Vitamin D"] | 3,026 |
|  | #2 | "Vitamin D":ti,ab,kw | 5,539 |
|  | #3 | [mh "Cholecalciferol"] | 1,674 |
|  | #4 | "Cholecalciferol":ti,ab,kw or "Calciol":ti,ab,kw or "(3 beta,5Z,7E)-9,10-Secocholesta-5,7,10(19)-trien-3-ol":ti,ab,kw or "Vitamin D 3":ti,ab,kw or "Vitamin D3":ti,ab,kw or "Cholecalciferols":ti,ab,kw | 1,853 |
|  | #5 | [mh "25-Hydroxyvitamin D 2"] | 64 |
|  | #6 | "25-Hydroxyvitamin D 2":ti,ab,kw or "25 Hydroxyvitamin D 2":ti,ab,kw or "25-Hydroxyergocalciferol":ti,ab,kw or "25 Hydroxyergocalciferol":ti,ab,kw or "25-Hydroxyvitamin D2":ti,ab,kw or "25 Hydroxyvitamin D2":ti,ab,kw or "9,10-Secoergosta-5,7,10(19),22-tetraene-3 beta,25-diol":ti,ab,kw or "Ercalcidiol":ti,ab,kw or "25-Hydroxycalciferol":ti,ab,kw or "25 Hydroxycalciferol":ti,ab,kw | 90 |
|  | #7 | [mh "Vitamin D Deficiency"] | 699 |
|  | #8 | "Vitamin D Deficiency":ti,ab,kw or "Deficiency, Vitamin D":ti,ab,kw or "Deficiencies, Vitamin D":ti,ab,kw or "Vitamin D Deficiencies":ti,ab,kw | 1,206 |
|  |  | "25-hydroxyvitamin D" [Supplementary Concept] |  |
|  | #9 | "25-hydroxyvitamin D":ti,ab,kw | 1,514 |
|  | #10 | [mh Avitaminosis] | 1,544 |
|  | #11 | "Avitaminosis":ti,ab,kw or "Avitaminoses":ti,ab,kw or "Deficiency, Vitamin":ti,ab,kw or "Deficiencies, Vitamin":ti,ab,kw or "Vitamin Deficiencies":ti,ab,kw or "Vitamin Deficiency":ti,ab,kw | 256 |
|  | #12 | [mh Calcitriol] | 710 |
|  | #13 | "Calcitriol":ti,ab,kw or "1 alpha,25-Dihydroxyvitamin D3":ti,ab,kw or "1 alpha,25 Dihydroxyvitamin D3":ti,ab,kw or "D3, 1 alpha,25-Dihydroxyvitamin":ti,ab,kw or "alpha,25-Dihydroxyvitamin D3, 1":ti,ab,kw or "1,25-Dihydroxyvitamin D3":ti,ab,kw or "1,25 Dihydroxyvitamin D3":ti,ab,kw or "D3, 1,25-Dihydroxyvitamin":ti,ab,kw or "1 alpha,25-Dihydroxycholecalciferol":ti,ab,kw or "1 alpha,25 Dihydroxycholecalciferol":ti,ab,kw or "1,25-Dihydroxycholecalciferol":ti,ab,kw or "1,25 Dihydroxycholecalciferol":ti,ab,kw or "Bocatriol":ti,ab,kw or "Leo Brand of Calcitriol":ti,ab,kw or "Calcitriol Leo Brand":ti,ab,kw or "Calcijex":ti,ab,kw or "Abbott Brand of Calcitriol":ti,ab,kw or "Calcitriol Abbott Brand":ti,ab,kw or "Calcitriol KyraMed":ti,ab,kw or "KyraMed, Calcitriol":ti,ab,kw or "KyraMed Brand of Calcitriol":ti,ab,kw or "Calcitriol KyraMed Brand":ti,ab,kw or "Calcitriol-Nefro":ti,ab,kw or "Calcitriol Nefro":ti,ab,kw or "CalcitriolNefro":ti,ab,kw or "Medice Brand of Calcitriol":ti,ab,kw or "Calcitriol Medice Brand":ti,ab,kw or "Decostriol":ti,ab,kw or "Jenapharm Brand of Calcitriol":ti,ab,kw or "Calcitriol Jenapharm Brand":ti,ab,kw or "MC1288":ti,ab,kw or "MC-1288":ti,ab,kw or "MC 1288":ti,ab,kw or "Osteotriol":ti,ab,kw or "Gry Brand of Calcitriol":ti,ab,kw or "Calcitriol Gry Brand":ti,ab,kw or "Renatriol":ti,ab,kw or "RenaCare Brand of Calcitriol":ti,ab,kw or "Calcitriol RenaCare Brand":ti,ab,kw or "Rocaltrol":ti,ab,kw or "Roche Brand of Calcitriol":ti,ab,kw or "Calcitriol Roche Brand":ti,ab,kw or "Hoffmann-La Roche Brand of Calcitriol":ti,ab,kw or "Hoffmann La Roche Brand of Calcitriol":ti,ab,kw or "Silkis":ti,ab,kw or "Galderma Brand of Calcitriol":ti,ab,kw or "Calcitriol Galderma Brand":ti,ab,kw or "Sitriol":ti,ab,kw or "Alphapharm Brand of Calcitriol":ti,ab,kw or "Calcitriol Alphapharm Brand":ti,ab,kw or "Soltriol":ti,ab,kw or "Tirocal":ti,ab,kw or "Cryopharma Brand of Calcitriol":ti,ab,kw or "Calcitriol Cryopharma Brand":ti,ab,kw or "20-epi-1alpha,25-dihydroxycholecaliferol":ti,ab,kw or "20 epi 1alpha,25 dihydroxycholecaliferol":ti,ab,kw or "1,25-dihydroxy-20-epi-Vitamin D3":ti,ab,kw or "1,25 dihydroxy 20 epi Vitamin D3":ti,ab,kw or "D3, 1,25-dihydroxy-20-epi-Vitamin":ti,ab,kw or "1,25(OH)2-20epi-D3":ti,ab,kw or "1 alpha, 25-dihydroxy-20-epi-Vitamin D3":ti,ab,kw or "1 alpha, 25 dihydroxy 20 epi Vitamin D3":ti,ab,kw | 1,434 |
|  | **#14 Combine** | **{or #1-#13}** | **7,903** |
|  | #15 | "frailty":ti,ab,kw | 477 |
|  | #16 | [mh "Frail Elderly"] | 629 |
|  | #17 | "Frail Elderly":ti,ab,kw or "Elderly, Frail":ti,ab,kw or "Frail Elders":ti,ab,kw or "Elder, Frail":ti,ab,kw or "Elders, Frail":ti,ab,kw or "Frail Elder":ti,ab,kw or "Functionally-Impaired Elderly":ti,ab,kw or "Elderly, Functionally-Impaired":ti,ab,kw or "Functionally Impaired Elderly":ti,ab,kw or "Frail Older Adults":ti,ab,kw or "Adult, Frail Older":ti,ab,kw or "Adults, Frail Older":ti,ab,kw or "Frail Older Adult":ti,ab,kw or "Older Adult, Frail":ti,ab,kw or "Older Adults, Frail":ti,ab,kw | 980 |
|  | **#18 Combine** | **{or #15-#17}** | **1,263** |
|  | **#19 Combine** | **#14 and #18** | **58** |
|  |  |  |  |
|  |  |  |  |
| **PubMed** | **Cochrane** | **Number of sum (Pubmed, EMBASE and Cochrane)** | **duplicates** |
| **292** | **58** | **350** | **147** |
