# Supplementary material for: Low 25-hydroxyvitamin D levels and the risk of frailty syndrome: a systematic review and dose-response meta-analysis
Source: BMC Geriatr. 2018 Sep 4;18:206. doi: 10.1186/s12877-018-0904-2 (PMC6124011; doi:10.1186/s12877-018-0904-2)
Supplement: Supplementary file 3 — Quality of the observational studies in the meta-analysis based on the Newcastle-Ottawa Scale. (DOCX 14 kb) [file 12877_2018_904_MOESM3_ESM.docx]

**Additional file 3: Quality of the observational studies in the meta-analysis based on the Newcastle-Ottawa Scale**

|  | Selection |  |  |  | Comparability | Exposure |  |  |  | |
| --- | --- | --- | --- | --- | --- | --- | --- | --- | --- | --- |
| Cross-sectional study | Case  definition  adequate | Representativeness of the cases | Selection of controls | Definition of  controls | Comparability of cases and controls | Ascertainment  of exposure | Same method of ascertainment. for cases and controls | Non-response  rate | Total score^*^ | |
| Puts et al., 2005 | 1 | 1 | 1 | 0 | 2 | 1 | 1 | 1 | 8 | |
| Ensrud et. al., 2010 | 1 | 1 | 1 | 0 | 1 | 1 | 1 | 0 | 6 | |
| Ensrud et al., 2011 | 1 | 1 | 1 | 0 | 1 | 1 | 1 | 0 | 6 | |
| Tajar et al., 2013 | 1 | 1 | 1 | 0 | 1 | 1 | 1 | 0 | 6 | |
| Schöttker et al., 2014 | 1 | 1 | 1 | 0 | 1 | 1 | 1 | 1 | 7 | |
| Pabst et al., 2015 | 1 | 1 | 1 | 0 | 2 | 1 | 1 | 1 | 8 | |
|  | Selection |  |  |  | Comparability | Outcome |  |  |  |  |
| Cohort study | Representativeness of the exposed Cohort | Selection of the non-exposed cohort | Ascertainment of exposure | Demonstrate that outcome of Interest was not present at start of study | Comparability of cohorts on the basis of the design or analysis | Assessment of outcome | Was follow-up long enough for outcomes to occur? | Adequacy of follow-up of cohorts | Total score | |
| Puts et al., 2005 | 1 | 1 | 1 | 1 | 2 | 1 | 0 | 1 | 8 | |
| Schöttker et al., 2014 | 1 | 1 | 1 | 1 | 1 | 1 | 1 | 1 | 8 | |
| Vogt et al., 2015 | 1 | 1 | 1 | 1 | 2 | 1 | 0 | 1 | 8 | |
| Buta et al., 2017 | 1 | 1 | 1 | 1 | 1 | 1 | 1 | 1 | 8 | |
