# Supplementary material for: Low 25-hydroxyvitamin D levels and the risk of frailty syndrome: a systematic review and dose-response meta-analysis
Source: BMC Geriatr. 2018 Sep 4;18:206. doi: 10.1186/s12877-018-0904-2 (PMC6124011; doi:10.1186/s12877-018-0904-2)
Supplement: Supplementary file 4 — Publication bias for association between serum 25-hydroxyvitamin D concentration per 25-nmol/L increment and frailty syndrome. Figure S1. Begg’s Funnel plot with 95% confidence intervals in the meta-analysis of the cross-sectional studies. Figure S2. Begg’s Funnel plot with 95% confidence intervals in the meta-analysis of the prospective cohort studies. (DOCX 59 kb) [file 12877_2018_904_MOESM4_ESM.docx]

**Additional File 4: Publication bias for association between serum 25-hydroxyvitamin D concentration per 25-nmol/L increment and frailty syndrome**

Figure S1: Begg’s Funnel plot with 95% confidence intervals in the meta-analysis of the cross-sectional studies.

**

**

Egger's test P =0.007

Figure S2: Begg’s Funnel plot with 95% confidence intervals in the meta-analysis of the prospective cohort studies.

**

**

Egger’s test: *P* = 0.693
